# Comparison of Current Immunotherapy Approaches and Novel Anti-Cancer Vaccine Modalities for Clinical Application

**DOI:** 10.3390/ijms26178307

**Published:** 2025-08-27

**Authors:** Elaine Meade, Mary Garvey

**Affiliations:** Department of Life Science, Atlantic Technological University, F91 YW50 Sligo, Ireland; elaine.meade@atu.ie

**Keywords:** vaccine, oncology, anti-cancer, neoantigens, immunotherapy, nucleic acid therapeutics

## Abstract

Despite improved diagnostic and treatment protocols, cancer remains a leading cause of morbidity and mortality globally. There are increasing rates of certain cancer types, including the highly drug-resistant colorectal cancer, in younger population cohorts. Therapeutic advances in oncology have led to the application of immunotherapy-based agents, including checkpoint inhibitors, antibodies, and adoptive cell therapies. Such immunotherapy approaches are greatly hindered by the tumour microenvironment and lack of specificity. Therapeutic vaccines are an innovative and rapidly advancing area of oncology, having potential for application as mono- and combined therapy in clinical settings, offering long term efficacy against disease recurrence. Advances in vaccine production using gene editing and bioprocessing techniques allows for novel vaccine types, including protein-based subunit vaccines, virus-like particle vaccines, and viral vector- and nucleic acid-based (RNA and DNA) vaccines. Cancer vaccines are designed to deliver specific tumour antigens, which activate anti-cancer cytotoxic T cells and helper T cells to produce immune memory, providing long term anti-cancer action. When coupled with advances in machine learning and artificial intelligence, anti-cancer vaccines may revolutionise oncology protocols and improve patient prognosis. This review aims to discuss current immunotherapy options in cancer treatment and recent advances in anti-cancer vaccine modalities.

## 1. Introduction

Cancer, or neoplasm, is a disease state having a multifaceted aetiology, high prevalence rate and high mortality rate. Uncontrolled cell division in mutated abnormal cells leads to tumour formation and expansion locally by invasion and systemically by metastasis following angiogenesis [1]. The World Health Organisation (WHO)’s cancer agency, the International Agency for Research on Cancer (IARC), states that the global burden of cancer is increasing based on its 2022 assessment of global prevalence data [2]. The 3 most common cancers globally are lung cancer, breast cancer and colorectal cancer (CRC), having case fatality rates of 18.7%, 6.9%, and 9.3%, respectively [3]. Importantly, 35 million cases of cancer are predicted in 2050, an alarming increase of 77% on the 20 million cases in 2022, associated with risk factors of obesity, alcohol consumption, smoking and environmental pollution [2] (Table 1). The incidence rate of both cervical cancer and CRC in young adults (<55 years) is increasing annually (1–2%), with CRC the leading cause of cancer death in men under 55 years [4]. Incidence rates also increased by 1–2% annually for cervical (ages 30–44 years) and CRCs (ages <55 years) in young adults [5]. CRC was the fourth-leading cause of cancer death in both men and women younger than 50 years in the late 1990s but is now first in men and second in women [6]. While prevalence rates are increasing, the survival rate of patients is also increasing due to advances in diagnostic techniques, early screening, and novel therapeutic approaches [7]. Treatment of cancer includes surgery, radiation, chemotherapy using anticancer active pharmaceutical ingredients (APIs), targeted therapy, hormonal and immunotherapy methods [4]. Studies show, however, an increased risk of cancer in adult survivors of childhood cancers such as Hodgkin lymphoma, where radiation therapy and the chemotherapeutic anthracycline increased the risk of breast cancer in 50-year-old female survivors of childhood cancer [8]. Increased rates of additional morbidities, including cardiomyopathy and heart failure, are also present [9], indicating iatrogenic comorbidities in cancer survivors. Additionally, iatrogenic menopause resulting from the treatment of cervical cancer is associated with increased rates of chronic morbidity and mortality in patients [10]. The occurrence of morbidities associated with treatment, such as iatrogenic carcinogenesis and malignancies, is a result of the toxicity of current treatment approaches, which induce cytotoxic and genetic damage in healthy cells due to non-specific cell targeting [11]. Issues with drug therapy, including drug pharmacokinetic profiles, adverse drug reactions (ADRs), drug resistance due to gene mutations and metabolic alterations, and drug selectivity, greatly hinder effective treatment protocols [12]. There is an urgent need for more effective and specific cancer treatment regimens to reduce mortality and improve the quality of life (QOL) of patients under treatment and safeguard patients from iatrogenic disease at a later stage. With this aim, novel therapeutic approaches under assessment include nanomaterial carrier systems e.g., polymeric and inorganic nanoparticles, checkpoint inhibitors (ICIs), chimeric antigen receptor (CAR) T cell therapy, T cell receptor (TCR) alterations, RNA-based therapies, and cytokine therapy [5]. More recently, novel immunotherapy methods, including cancer vaccines such as protein-based, cell-based, nucleic acid-based and viral vaccines [13,14], are being investigated [15]. Immunotherapy approaches aim to overcome the limitations of standard therapies by providing a more targeted and specific method of eliminating cancer cells by stimulating the patient’s own immune system to destroy cancer cells while safeguarding healthy cells, thereby reducing ADRs. Cancer vaccines may offer effective alternative standalone or combined treatment options, providing improved QOL and mortality rates for oncology patients. This review discusses the current advances in the therapeutic cancer vaccines, outlining their efficacy and clinical limitations in comparison to other immunotherapy approaches.

## 2. Tumorigenesis and Immunotherapy Approaches

Cancer is a genetic disease resulting from the accumulation of mutations in key cell cycle genes (proto-oncogenes) which can be inherited (germline mutation) or result from exposure to carcinogens, as per the Somatic Mutation Theory (SMT). This mutational activation of oncogenes allows for the initiation of carcinogenesis (initiation of a tumour) and tumorigenesis [29]. Mutations such as point mutations or chromosomal aberrations follow exposure to mutagenic agents such as chemicals, ultraviolet radiation (UV), or oncogenic viruses, e.g., Human papilloma virus (HPV). If DNA repair mechanisms are not effective or operational (due to genetic mutations), these mutations can enter the cell cycle, leading to carcinogenesis. Mutation in driver proto-oncogenes, including the RAS, KRAS, and BRAF genes, amongst others, leads to unregulated cell division and apoptosis evasion [30]. The mutated cells have a clonal advantage, allowing for cell proliferation and the accumulation of further genetic and epigenetic changes, resulting in an irreversible heterogeneous tumour capable of tissue invasion and angiogenesis [31]. Mutations resulting in the inactivation of tumour suppressor genes, e.g., TP 53, BRCA 1 and 2, and XPA, promote tumour progression [32]. Inactivation of tumour suppressor action is highly prevalent in many cancers, including ovarian, lung, CRC, pancreatic, and breast cancer, amongst others [33]. Immune evasion or resistance is also an important aspect of tumour progression and metastasis, where neoantigens (highly specific antigens) on cancer cells are key players in immune cancer recognition [34]. Studies describe the role of neoantigens in cancer immunotoxicity due to their involvement in T-cell responses mediated by antigen-presenting cells (APCs), dendritic cells [35] which present antigen peptides to cytotoxic T-cells (CD8+ cells) via the major histocompatibility complex (MHC). Killer T cells are essential in fighting cancer, as they possess the ability to bind these antigens on the tumour cells and have become the focus of immunotherapy treatment. Cancer cells can evade this immune response by downregulating or not expressing TAAs and TSAs, due to mutations in related genes. Therefore, the tumour evades anti-cancer immune responses by excluding immunogenic antigens or producing cells free of antigens which cannot be recognised by T cells. Additionally, by making an immunosuppressive tumour microenvironment (TME) where cancer cells avoid immune attack and by stimulating T cell exhaustion [36]. T cell exhaustion and inhibition of cytotoxic T cell activity in the TME are achieved by the activation of inhibitory immune checkpoint pathways [37]. Tumours inhibit T cells by reducing the expression of co-stimulatory factors and MHC, thereby reducing T cell stimulation [38]. The TME is greatly impacted by metabolic changes and associated stress due to heterogeneity, lack of oxygen, and nutrients in a highly metabolically active area creating pH alterations [39]. In hypoxic areas cancer cells often secrete lactate as a byproduct of aerobic glycolysis, contributing to the energy needs of reproducing cancer cells. Increased lactate levels have an inhibitory effect on CD8+ and natural killer (NK) cells and support immunosuppressive T reg cells, thereby contributing to the immune evasion in the TME [39]. An excellent review of the TME in oncology is provided elsewhere [40]. In the TME, immunosuppressive IL-10 and prostaglandin E2 are produced, which are involved with apoptosis of CD8^+^ T cells infiltrating the tumour [38]. Currently, the TME is the focus of most immunotherapy research with the aim of enabling an effective immune response in this immunosuppressive environment.

Unregulated proliferation of cells carrying mutated genes results in tumorigenesis, which can remain local (termed benign) or spread systemically through metastasis, in which case it is considered malignant [1]. Certainly, the malignancy of the tumour largely determines the prognosis. Cancer is categorised as carcinoma affecting epithelial cells, sarcomas affecting connective tissues and leukaemia and lymphomas affecting blood cells and further grouped according to tissue of origin, such as breast, liver, etc. Ultimately, tumours develop in tissues or organs which have lost cellular homeostasis and have hyperplastic (increase in cell number), dysplastic (abnormal cell types) or regenerative changes, where risk of cancer progression is associated with increasing grades of dysplasia [41] with increasing prevalence globally [42].

### 2.1. Limitations of Traditional Oncology Approaches

Traditional cancer treatment approaches, including surgery of resectable tumours, chemotherapy, including small molecules, hormone therapy, and radiation therapy are applied to oncology patients. Depending on the cancer type, stage of disease and trophism, therapeutics are chosen based on optimal efficacy and delivered locally and/or systemically with surgical removal if possible [5,43,44]. The pharmacokinetic and pharmacodynamic profile of chemotherapeutics varies and is hindered by drug resistance [45], the acidic TME, low specificity for cancer cells over healthy cells, tumour location, and receptor availability for therapeutic agonists or antagonist, e.g., tamoxifen for oestrogen-positive breast cancer [46]. For example, the drug therapies applied in CRC, including 5-fluorouracil, oxaliplatin (associated with peripheral neuropathy), capecitabine, and irinotecan, are cytotoxic to healthy cells, producing ADR, where resistance to fluorouracil is prevalent in most cases, having a response rate of <10% [47]. Similarly, fluorouracil, oxaliplatin, and irinotecan are administered in the treatment of prostate cancer; however, high rates of resistance are evident, resulting in a 5-year survival rate of just 13% [48], the lowest survival rate among all cancers [6]. In drug therapy, there are potential issues with interindividual variability in API pharmacodynamics due to polymorphism in genes coding for key liver enzymes involved with the pharmacokinetics of the active drug. For example, alterations in cytochrome P450 isoforms, e.g., the CYP2D6 gene and enzyme, impact the liver’s biotransformation of tamoxifen to the active endoxifen in vivo, which negatively affects patient outcome [49]. Importantly, pharmacogenetic variations such as polymorphisms in key enzymes are also involved in the formation of iatrogenic malignancies due to alterations in drug metabolising enzymes (e.g., glutathione-S-transferases) and DNA repair enzymes (e.g., BRCA2 (FANCD1) and BRIP1 (FANCJ)) [11]. The ADRs and iatrogenic diseases, including secondary malignancies associated with chemotherapy and radiation treatment, often reduce the QOL of patients with variable impact on their cancer prognosis [12]. Studies show that oncology patients have high rates of comorbidity and polypharmacy, with >21% of admissions to oncology wards due to ADRs from systemic anticancer therapies (SACTs), opioids, corticosteroids, and nonsteroidal anti-inflammatory drugs (NSAIDs) [50]. Consequently, novel oncology approaches are needed to improve mortality rates and patient QOL by overcoming these limitations. Oncology and cancer treatment are currently undergoing a paradigm shift towards innovative methods, including more personalised approaches. Therapies based on immunotherapy, e.g., ICIs, cytokines, CAR T cells treatment, and personalised cancer vaccines, are at the forefront of research. Such approaches stimulate the patient’s own immune system to selectively target and destroy cancer cells while safeguarding healthy cells, thereby reducing ADRs.

### 2.2. Immunotherapy Modalities to Overcome Traditional Therapy Limitations

There has been a surge in cancer treatment based on immunotherapy in the last two decades, with checkpoint inhibitors, bispecific antibodies (BsAbs), cytokines, adoptive cell therapies (ACTs), and more recently oncolytic viruses and vaccines applied in oncology protocols [38]. Tumour-infiltrating lymphocyte (TIL) therapy and CAR T-cell therapy are the two main types of ACT using a patient’s own immune cells to attack cancer cells. TIL involves isolating T cells which have infiltrated the tumour, culturing and expansding ex vivo with re-infusion into the patient, where they attack cancer cells, both within the tumour and in the blood system [51,52,53]. The FDA approved lifileucel (Amtagvi), a TIL-based treatment for the treatment of melanoma [52]. The studies of Tran et al. (2016) report the efficacy of ACT, specifically T cells targeting *KRAS* mutations from metastatic CRC acting as TILs in the treatment of lung metastases [54]. CAR T-cell therapy involves isolating T cells from the patient’s peripheral blood system, genetically modifying the T cells by adding the CAR, expanding and culturing the modified cells, and then re-infusing them into the patient as treatment. These engineered CAR T cells exert a cytotoxic effect on cancer cells expressing specific neoantigens [53]. CAR T-cell therapy has proven effective in the treatment of blood cancers. ICIs are monoclonal antibodies (mAbs) that target inhibitory checkpoint molecules which are expressed by APCs and CD4^+^ cells [55]. Programmed-death ligand-1 (PDL-1) is applied as a biomarker in selecting patients for treatment with specific ICIs. PDL-1 limits activation of T cells, decreasing the immune response to cancer cells [55]. ICIs induce immune-related toxic effects by stimulating T cells to damage healthy host tissue, which commonly affects the colon, liver, skin, and pituitary gland, which may be fatal [56]. Nivolumab, a fully human anti–PD-1 antibody, and ipilimumab, a fully human anti–cytotoxic T-lymphocyte antigen 4 (CTLA-4) antibody, are both ICIs demonstrating complementary action in melanoma patients and renal cell carcinoma [57]. The tumour mutational burden (TMB) impacts on the efficacy of ICIs, with higher TMB and low immunosuppression (hot tumours) increasing efficacy. The TMB levels, however, vary with tumour type, and in cancer patients, e.g., renal cancer, ovarian, and breast cancers have intermediate TMB levels, with paediatric tumours and leukaemias having a lower TMB [55]. The higher TMB associated with melanoma and lung cancer makes them more susceptible to the effects of ICIs [57]. mAbs are immune components produced via bioprocessing which specifically target cell surface antigens that are displayed ectopically on cancer cells, allowing for enhanced specificity in treatment; examples include Margetuximab for breast cancer and Isatuximab for myeloma [58]. Sintilimab is a PD-1 blocking antibody produced via yeast display technology approved for the treatment of Hodgkin’s lymphoma [59]. mAbs are used in combination with traditional chemotherapeutic approaches, where they enhance the activity of chemotherapeutics and radiation therapy. An excellent review of clinical mAb is provided by Al-Taie et al. (2024) [60]. BsAbs (targeting 2 antigens) and trispecific mAbs allow for improved targeted specific efficacy, with BsAbs (e.g., blinatumomab) applied in the treatment of haematologic cancers [61]. The combining of antibodies to small molecule APIs has led to the emergence of antibody drug conjugates (ADCs), which selectively deliver a potent API directly to the tumour, inducing cytotoxicity. Tisotumab vedotin (Tivdak™) is an ADC consisting of a fully human mAb conjugated to monomethyl auristatin E (MMAE) given accelerated approval by the FDA for the treatment of metastatic cervical cancer unresponsive to standard chemotherapy [62]. Sacituzumab govitecan approved in 2021 by the FDA for the treatment of unresectable triple-negative breast cancer [63]. More recently, the FDA approved a clinical study investing a bispecific “OR-Gate” autologous CAR T-cell therapy targeting the B-cell antigens CD19 and CD20 (IMPT-314) with completion due in 2029 [64]. Cytokines, which are immune regulators, are applied in cancer therapy to activate the immune system of cancer patients where chronic low-grade inflammation is believed to contribute to cancer aetiology [65]. Cytokines, including interleukin-2 (IL-2), IL-12, IL-15, IL-18, IL-21, granulocyte-macrophage colony stimulating factor (GM-CSF), CCL21 and type 1 interferons (IF), demonstrated antitumour activity in preclinical studies [66]. Recently, in 2024, the FDA approved the interleukin (IL)−15 superagonist, N-803 (Anktiva, nogapendekin alfa inbakicept-pmln), for the treatment of bladder cancer [67]. IFN alpha and high doses of IL-2 are approved for adjuvant treatment of completely resected high-risk melanoma cancer amongst other refractory cancers and metastatic renal cell cancers and melanoma, respectively [68].

### 2.3. Limitations of Immunotherapies

For many immunotherapy approaches, treatment has only proven successful in a portion of malignancies, primarily due to factors such as cancer heterogeneity, stage of cancer, patient immunogenicity, lack of more specific biomarker profiles, and the variable TME [69] (Table 2). Clinical application of such immunotherapies is associated with cytokine release syndrome (CRS), immune effector cell-associated neurotoxicity syndrome (ICANS), infusion-related reactions (IRRs), autoimmunity, and opportunistic infections [70]. The application of cytokines is limited, for example, by their low efficacy, serious ADRs (e.g., cytokine storm), short half-life in vivo, and off-target immunoregulation influences [71]. Limitations to broad-scale use of mAbs and their derivatives include their ADR profile, production limitations, pharmacokinetic profiles, and high cost or patient accessibility. Data shows that while ca. 43% of oncology patients are suitable for ICI therapy, approximately 12% respond positively to treatment [72]. ICIs are associated with ADRs, including fatigue, diarrhoea, immune dysregulation and neutropenia [57]. Additionally, acquired resistance to ICIs hinders application [73]. TIL therapy is limited by challenges in obtaining enough TILs, which are not always present in tumours. Moreover, TILs are often exhausted or impaired within the TME, may lack specificity for tumour antigens, and are impacted by the presence of the MHC [51]. CAR T cells are hindered by the TME, T cell exhaustion and an inability to infiltrate and penetrate solid tumours, limiting their application in oncology to blood cancers [53]. Clinical trials assessing the efficacy of CAR T cells against solid tumours when administered via alternative routes are being conducted, namely for glioblastoma (NCT02208362) and leptomeningeal metastases (NCT03696030) [74]. Importantly, CAR T cells can be down-regulated by the TME and inhibitory cytokines or by modulating effects induced by stromal cells [75]. The gene editing technology clustered regularly interspaced short palindromic repeats (CRISPR)-associated protein (CRISPR-Cas9) has been applied to CAR T cells to improve efficacy, potency and safety. The research of Stadtmauer et al. (2020) used CRISPR to remove the endogenous T cell receptor (TCR) and the immune checkpoint molecule PD-1 to improve the cytotoxic antitumour effect of engineered T cells [76]. Additional gene editing tools include zinc-finger nucleases (ZFNs) and transcription activator-like effector nucleases (TALENs), which are not as flexible, multiplex or efficient as CRISPR techniques [75]. Anti-cancer vaccine therapies aim to overcome the limitations of current immunotherapy protocols by providing a more specific targeting of tumour cells which is not hindered by the TME while having high levels of immunogenicity and biocompatibility (Table 2). In anti-cancer vaccine development, selecting the ideal antigen is key to specific target therapy; such antigens should be expressed on cancer cells, aid in preventing cancer immune escape, and have limited expression on healthy cells [77].

## 3. Anti-Cancer Vaccine Modalities

Therapeutic vaccines are an innovative and rapidly advancing area of oncology utilising novel vaccine modalities as preventative and therapeutic anti-cancer therapy. They have potential for application as mono- and combined therapy in clinical settings, offering long term efficacy against disease recurrence. Advances in vaccine production using gene editing and bioprocessing techniques allow for novel vaccine types, including protein-based subunit vaccines, virus-like particle (VLP) vaccines, and viral vector- and nucleic acid-based (RNA and DNA) vaccines [59]. Cancer vaccines are designed to deliver tumour antigens or their genes to APCs, which, when processed, activate anti-cancer cytotoxic T-cell and helper T-cell (CD4+ cells) responses and produce immune memory to provide long term anti-cancer action [92,93]. These activated T cells then initiate the death of tumour cells. To boost immune responses, cancer vaccines can be formulated containing adjuvants to augment or direct the specific immune response to a tumour antigen.

### 3.1. Preventative Vaccines

Preventative or prophylactic anti-cancer vaccines include the human papillomavirus (HPV) and hepatitis B virus (HBV) vaccines, which are causative of cervical and liver cancer, respectively. The incidence rate of cervical cancer in young adults (<55 years) is increasing annually (1–2%) [4], with ca. 90% of cases associated with the oncogenic HPV viruses, with HPV 16 and 18 causing 71% of cases [94]. Additional strains associated with cervical cancer include HPV 45, 31, 33, 52 and 58. The virus infects the epithelial cells of the cervix, where persistence and immune evasion results, in cervical intraepithelial neoplasia (CIN), with severity graded on a scale of CIN 1 (mild), 2 (moderate), and 3 (severe or high-grade) dysplasia [95]. Studies indicate that 30% of untreated CIN 3 cases will result in cervical cancer over 8 to 15 years [96]. Importantly, HPV-associated cancers of the vagina, head and neck, throat and oropharyngeal squamous cell carcinoma are also increasing [97]. HPV vaccines are formulated using antigenic structural proteins of the HPV virus, namely L1, in the form of virus-like particles (VLPs) [98]. VLPs typically have multiple subunits and resemble the viral structure without the genetic material present, allowing for strong immunogenicity with no risk of pathogenicity [93]. VLPs are engineered as prophylactic anti-cancer agents by producing a humoral immune response in vivo and activating CD8+ cells and CD4+ cells which triggers B cells and macrophages [99]. VLPs are produced in bioreactors using different biological expression systems, e.g., *Escherichia coli* is used to produce Cecolin^®^, and *Saccharomyces cerevisiae* and *Pichia pastoris* are used to produce Gardasil^®^ [59]. At present, there are six licensed HPV vaccines recommended by the WHO for immunisation against HPV: three bivalent (Cecolin^®^, Cervarix™, and Walrinvax^®^), two quadrivalent (Gardasil^®^ with Cervavac^®^ being nationally licensed), and one nonavalent vaccine (Gardasil 9) [100]. Bivalent vaccines are immunisations against 2 HPV strains e.g., Cervarix against HPV 16 and HPV 18; quadrivalent vaccines are against 4 HPV strains e.g., Gardasil against HPV 6, 11, 16, and 18; and nonavlent vaccines are effective against 9 HPV strains [94]. The global HPV vaccination programme has the aim of preventing cervical cancer as part of a foundational pillar of the WHO Global Strategy to Accelerate the Elimination of Cervical Cancer as a Public Health Problem [100]. Cecolin, a single-dose bivalent vaccine against HPV 16 and 18, was announced by the WHO in 2024, having demonstrated safety and immunogenicity in clinical trials and was not inferior to Gardasil [98]. A single-dose vaccine is desirable to increase uptake in low- to middle-income countries (LMICs) where multi dosing regimens are a barrier to immunisation [101]. Studies demonstrate the significant reduction in cases of cervical cancer in women since the application of HPV vaccines in adolescents, with herd protection also evident in unvaccinated women [102]. The mass vaccination of girls aged 12–13 in the United Kingdom with the bivalent vaccine Cervarix significantly reduced (97%) the incidence of CIN 3 and cervical cancers nationally [103]. Gardasil-9, produced by Merck, is a nine-valent HPV vaccine against HPV 6, 11, 16, 18, 31, 33, 45, 52, and 58 [94]. Studies report an efficacy of 97% against HPV 16 and 18 using a single dose of a bivalent and nonvalent vaccines in girls aged 15–20 years and 88% efficacy against additional oncogenic strains of HPV 16, 18, 31, 33, 45, 52, or 58 [101]. Additional studies verify that a single dose of HPV vaccine gives comparable immunogenicity against HPV 16 and 18 morbidity and carcinogenesis across different geographical locations and population cohorts [104].

Chronic HBV infection is associated with up to 90% of hepatocellular carcinomas (HCC) in adults and ca. 100% of childhood cases in endemic areas [105]. The FDA has licensed three single-antigen vaccines (Engerix-B, Heplisav-B, and Recombivax HB) and three combination vaccines (Pediarix, Vaxelis, and Twinrix) for immunisation against HBV [106]. Vaccines against HBV as a method of HCC prevention have been implemented for ca. 30 years, resulting in an 80% reduction in disease prevalence [107]. These VLPs vaccines are composed of the immunogenic small surface antigen (HBsAgS) of the HBV produced in yeast expression systems [108]. The HBV vaccine provides 72% protection against liver cancer and 64% protection against associated mortality when given to infants up to 6 months old [109]. Palladini et al. (2018) developed a VLP to treat human epidermal growth factor receptor-2 (HER2)-positive breast cancer expressing SpyCatcher-HER2 fusion antigen [110].

HBV-derived VLPs are being investigated in the production of vaccines against *Helicobacter pylori* (*H. pylori*) bacteria, which is associated with stomach cancer, hepatitis C virus (HCV), HPV-associated cancers, and four serotypes of dengue virus [108]. The anti-malaria vaccine RTS, S is a VLP using malaria sporozoite antigen circumsporozoite protein from Plasmodium falciparum on an HBV scaffold that was immunogenic in test mice, generating a strong immunoglobulin G response [111]. The RTS, S vaccines were recommended by the WHO to prevent malaria in children in Africa in October 2021, with R21/Matrix-M vaccines also recommended in 2023 [112]. This is important, as data shows that the malaria pathogen is associated with Burkitt lymphoma in children of LMICs [113]. Additionally, VLPs for influenza, rotavirus, Zika and HIV are currently in clinical trials [93].

### 3.2. Therapeutic Vaccines

Therapeutic cancer vaccines are treatment options applied after tumour development (unlike preventative vaccines) which aim to induce a potent cellular immune response which targets and eliminates existing cancer cells while also establishing a long-lasting immune memory to prevent cancer recurrence. They achieve this therapeutic effect by modulating the patient’s immune system to induce or enhance antitumour activity in vivo via directed immune stimulation, e.g., cytotoxic T cell activation [114]. These vaccines are grouped as cell-based including adoptive T-cell transfer or antigen-based vaccines, which aim to stimulate patient humoral response via the introduction of tumour antigens [35]. Types of therapeutic vaccines include nucleic acid-based vaccines (mRNA and DNA vaccines using tumour-associated antigens (TAAs) and tumour-specific antigens (TSA)s or neoantigens), oncolytic viruses, peptide-based, and dendritic cells (DCs) [115] (Table 3).

#### 3.2.1. Tumour Antigens Used in Vaccines

mRNA vaccines contain synthetic mRNA coding for TAAs or TSAs, while DNA vaccines contain plasmids of DNA sequences coding for antigenic proteins. The production and mode of action of vaccine types are described elsewhere [93,115]. Neoantigens are newly formed antigens generated and expressed by tumour cells, making them highly specific due to the genetic mutations and post-translational alterations present within tumour cells [116,117]. The use of TSA vaccines to induce tumour-specific immune responses has demonstrated safety and potential efficacy against urothelial cancer or renal-cell carcinoma and allows for a personalised treatment approach [118]. TAAs are, therefore, autoantigens expressed in normal tissues and overexpressed in various cancers. As such, neoantigens are individual-specific, offering a personal vaccine therapeutic approach which also, however, raises limitations in terms of difficulty, feasibility and cost [117]. Mesothelin (MSLN), for example, is a cell surface glycoprotein present on many malignant tumours, including lung, pancreatic, and ovarian cancer [119]. TAAs are non-mutated and are self-antigens, and are therefore, less specific immunotherapy targets where T cell tolerance limits TAA vaccine efficacy in clinical trials [120]. Initial cancer vaccines used TAAs (e.g., gp100 or MUC1), which are present on some healthy cells and tumour cells and lead to weak immunogenicity due to pre-existing tolerance to these antigens [121]. TAA vaccines are not considered a personalised approach due to their non-specific presence on tumours reducing specificity and are applied for general cancer vaccine therapy clinically [122].

#### 3.2.2. Nucleic Acid Vaccines

mRNA vaccines produce low immunogenicity in patients due to ineffective mRNA expression and subsequent poor immune stimulation, which limits their efficacy in oncology [123]. The research of Fournier et al. (2025) developed a nanostructured lipid carrier platform called Lipidots^®^ to deliver mRNA vaccines encoding Ovalbumin antigen as immunotherapy [124]. BNT111 is an mRNA-based cancer immunotherapy agent using 4 TAAs associated with melanoma, demonstrating antitumour activity as a standalone and combined therapy (with ICIs PD-1) in unresectable melanoma in phase I clinical trials [120]. A recent phase I clinical trial investigating the neoantigen vaccine Autogene cevumeran (BNT122), in combination with Atezolizumab (anti-PD-L1 mAb) and mFOLFIRINOX, T cell response and antitumour activity in resected pancreatic cancer patients [118]. The research of Guo et al. (2024) investigated a novel mRNA compartmentalisation-based cancer vaccine which demonstrated a strong immune response inhibiting tumorigenesis and metastasis [123]. Clinical studies assessing the safety and immunogenicity of an mRNA Epstein-Barr virus vaccine for the treatment of nasopharyngeal carcinoma have demonstrated limited efficacy, which was enhanced when applied in combination with adoptive NK cells [125].

DNA-based vaccines have many advantages over mRNA vaccines, as described elsewhere [126]. DNA vaccines are greatly limited; however, by their poor immunogenicity and poor cellular spread in vivo [127]. Mammaglobin-A DNA vaccine (NCT00807781) is a DNA vaccine in a phase I clinical trial for breast cancer which provided preliminary evidence of biologic efficacy and an improved overall survival in patients [128].

#### 3.2.3. Whole Cell Vaccines

DC vaccination methods typically involve the isolation of DCs with modification to express TSAs, expansion ex vivo and re-administration in vivo to induce antitumour immune responses [121]. This vaccination modality has limitations; however, including a short ex vivo half-life, antigen degradation, lack of cell therapy facilities with standardised methods and the high costs associated with production and expansion of cells [122]. Allogeneic whole cell vaccination has advantages over other modalities, including the use of many antigens and interaction with MHC I and II epitopes, which induce CD4+ and CD8+ activation [116]. ACT targeting p53 mutations, which are present in most cancers, induced a long-lasting response in patients having solid tumours [116]. Sipuleucel-T is an FDA-approved autologous cellular immunotherapy consisting of cultured peripheral blood mononuclear cells used to treat metastatic prostate cancer. The IMPACT phase III study shows that treatment with sipuleucel-T reduced the relative risk of prostate cancer by 22% [129]. Four other prostate cancer vaccines have reached phase III trials, namely GVAX (prostate cancer variant) containing irradiated prostate cancer cells, PPV peptide vaccine, PCVAC/PCa dendritic cell-based vaccine and PROSTVAC anti-PSA (prostate-specific antigen) vaccine [130]. The vaccine PROSTVAC contains PSA, which induces a T cell response capable of infiltrating tumours, which may be optimised when used combined with ICIs [131]. Studies show, however, no survival benefit of PROSTVAC compared to placebo in phase III trials [130].

#### 3.2.4. Oncolytic Viruses

Oncolytic viruses (OVs) are an emerging area of oncology therapeutics demonstrating promising efficacy in preclinical and clinical studies where they selectively infect cancer cells, reducing ADRs in patients [132]. OVs cause cell lysis, which releases TAAs and TSAs, viral pathogen-associated molecular patterns (PAMPs), and cellular damage-associated molecular patterns (DAMPs), which promote a local antitumour effect and can carry transgenes [13]. T-VEC was the first oncolytic viral therapy approved for the treatment of metastatic melanoma using adenovirus as the viral vector [13]. T-VEC is currently being assessed as a combined therapy with ICIs, e.g., ipilimumab and pembrolizumab, for systemic visceral metastases [15]. T-VEC uses a herpes simplex virus to specifically target and destroy cancer cells of unresectable melanoma skin cancer. The virus selectively replicates in tumours, producing granulocyte macrophage colony-stimulating factor, T-cell immune response and fatal cell lysis [13]. Imlygic^®^ is an oncolytic immunotherapy approved by the Food and Drug Association (FDA) in 2015 for the local treatment of unresectable cutaneous, subcutaneous and nodal lesions in recurrent melanoma patients having a tolerable side-effect profile [14]. The research of Sato-Dahlman et al. (2025) developed an oncolytic adenovirus targeting MSLN having selective and potent antitumour activity in vivo [133]. The adenovirus 5 is used as a viral vector delivery system in cancer vaccines, as it can hold and deliver many transgenes [121]. OVs’ efficacy in vivo is hindered by the TME, tumour heterogeneity, the MHC I, pharmacokinetics such as distribution and clearance, and pre-existing exposure to viral vectors such as the commonly used adenovirus, where antibodies are already present to the vector system [133]. Adenovirus type 5 is also associated with hepatotoxicity due to organ trophism following IV administration and systemic toxicity [134]. The studies of Ottensmeier et al. (2023) investigated a personalised cancer vaccine based on a Modified Vaccinia Ankara (MVA) viral vector to treat head and neck squamous cell carcinoma (HNSCC), which induced T cell responses and was safe and tolerable in clinical trial NCT04183166 [135].

**Table 3:** Platforms and technologies in cancer vaccine development.

Vaccine Platform	Mechanism of Action	Key Clinical Developments ^1^	Advantages & Limitations
mRNA-Based Vaccines	mRNA molecules encoding TAAs or viral antigens are delivered into host cells, where they are translated into proteins that elicit targeted immune responses.	Autogene cevumeran (BioNTech/Genentech): mRNA-Based Neoantigen Vaccines, Ongoing Phase I for locally advanced or metastatic tumours (NCT03289962) and Phase II for PDAC (NCT05968326); Preliminary results indicate specific and long-lasting CD8+ T cell immunity in PDAC, which is associated with a delayed recurrence in patients [136]. mRNA-4539 (Moderna/Merck): mRNA-Based Neoantigen Vaccines, Ongoing Phase II/III with Pembro for RCC (NCT06307431), SCC (NCT06295809) and melanoma (NCT05933577). Early results show reduced risk of recurrence and mortality in stage III/IV melanoma patients’ post-resection, with a hazard ratio of 0.56 (95% CI, 0.31 to 1.08, p. = 0.0266) [137]. WGc-043 (Walvax/Genentech): Vaccine targeting EBV-associated cancers (NCT06788600), early-phase trials ongoing. BNT111 (BioNTech): Ongoing Phase II trial for anti-PD-1-refractory, unresectable Stage III/IV melanoma, being evaluated alone or in combination with cemiplimab (NCT04526899). mRNA-2752 (Moderna): Lipid nanoparticle-encapsulated vaccine encoding OX40L, IL-23, and IL-36γ for intratumoural injection. Ongoing early trials in advanced malignancies, including TNBC, HNSCC, NSCLC, lymphoma, and high-risk DCIS, assessing safety and efficacy in combination with ICIs (NCT03739931, NCT02872025).	Rapid development enabled by synthetic mRNA technology; scalable manufacturing; strong antigen expression; adaptability to personalised neoantigens. They offer high tumour specificity, favourable safety profile due to non-integrating nature, and broad design flexibility. Limitations include cold-chain logistics, inflammatory side effects, and variable immunogenicity in patients, especially those with immune suppression or unfavourable tumour microenvironments [115,138,139].
DNA-Based Vaccines	Plasmids (circular DNA) or other DNA constructs encoding TAAs or viral antigens are delivered into the body (often via electroporation), where they enter host cells and express the encoded antigen, triggering an immune response.	pTVG-HP (Transgene): Ongoing phase II trials; NCT04090528 combines with pTVG-AR and Pembro, targeting mCRPC. NCT03600350 combines with Nivo and GM-CSF in patients with non-metastatic PSA-recurrent PCa. Early results show safety, clinical benefit (PSA decline in 21% of patients), and immunological activity, but no complete disease eradication [140]. WOKVAC (University of Wisconsin): Phase I in HER2-negative BC (NCT02780401) showed safety and immunogenicity [141]. Ongoing Phase II include NCT04329065 (combination with chemo and HER2-targeted mAbs) and NCT03384914 (HER2-positive BC recurrence prevention). STEMVAC (Vaxine): Multi-antigen polyepitope plasmid vaccine. Phase I in advanced BC (NCT02157051) showed selective Type I T-cell responses [142]. Ongoing Phase II trials: NCT05455658 (early-stage TNBC) and NCT05242965 (metastatic NSCLC with Pembro). INO-5401/INO-9012 (Inovio): Ongoing Phase I for BRCA1/2 mutation carriers (NCT04367675) and Phase I/II combining with ICIs for urothelial carcinoma (NCT03502785) and GBM (NCT03491683). Interim results in GBM show promising immune responses, acceptable safety, and potential survival benefit when combined with cemiplimab and RT [143]. VGX-3100 (Inovio): Vaccine targeting HPV-16 and HPV-18; Ongoing Phase II investigating its use in HIV-positive patients with high-grade anal lesions (NCT03603808). Phase III trials completed for CIN2/3 (NCT03185013), demonstrating histopathological regression (23.7% in the vaccinated group) and HPV clearance in Phase II trials [144,145].	Excellent stability, ease of manufacturing, and safety profiles [146]. They can be stored without cold-chain requirements and enable multivalent antigen design [146], which can broaden immune responses while also offering tumour specificity and scalability. However, they often require advanced delivery methods (e.g., electroporation) to achieve sufficient transfection efficiency and typically elicit lower immunogenicity compared to mRNA or viral vector platforms, with adjuvants or ICIs offering potential solutions to enhance efficacy [126]. Challenges with antigen presentation and immunosuppressive TME can limit effectiveness.
Peptide-Based Vaccines	Short AA sequences derived from TAAs or viral proteins are delivered with adjuvants to enhance immune activation and stimulate antigen-specific T cells to target cancer cells.	EVX-01 (Evaxion): Peptide-Based Neoantigen Vaccines: AI-designed vaccine with Pembro in Phase II for melanoma (NCT05309421); 67% response in Phase I [138]. Vaccine with Poly-ICLC and ICI (Seqker Biosciences): Peptide-Based Neoantigen Vaccines: Phase I for solid tumours (NCT06614140); targeting personalised neoantigens with immune modulation. SurVaxM (MimiVax): Ongoing Phase I for relapsed or progressive paediatric medulloblastoma, high-grade gliomas (including GBM),ependymoma, and newly diagnosed DIPG (NCT04978727). Phase II ongoing for newly diagnosed GBM (NCT05163080) and GBM with TMZ (NCT02455557); early signs of immune response and safety [147]. ELI-002 (Elisra Biotech): Multi-peptide vaccine targeting KRAS mutations and other TAAs in pancreatic and CRC; Phase I (NCT04853017) and Phase I/II (NCT05726864) trials ongoing; early results show improved immune activation with mKRAS-specific T-cell responses and tumour biomarker reduction [148]. Mutant KRAS-Targeted Long Peptide Vaccine (Sidney Kimmel Comprehensive Cancer Center at Johns Hopkins): Phase I (NCT05013216) for patients at high risk of pancreatic cancer or with pancreatic cystic neoplasms; investigating immune activation and cancer prevention through KRAS-targeted peptides. Galinpepimut-S (Sellas Life Sciences): Vaccine targeting WT1. Phase I in ovarian cancer (NCT02737787) showed promising immune responses, with a 70% 1-year PFS rate when combined with Nivo [149]. Phase III for AML maintenance therapy (NCT04229979) is active but not recruiting yet (as of the latest update). For information on Peptide-Based Neoantigen Vaccines, please refer to the Neoantigen Vaccines section.	Highly specific, targeting defined tumour antigens with a favourable safety profile. They can be manufactured quickly and cost-effectively. However, they often require adjuvants for optimal immunogenicity and may exhibit limited efficacy in patients with immune tolerance or low antigen expression. Additionally, challenges with antigen presentation and TME can hinder their ability to induce robust immune responses [150].
Dendritic Cell Vaccines	Autologous dendritic cells are isolated, loaded with tumour antigens (often neoantigens or TAAs), and reintroduced into the patient to stimulate a targeted T cell response against cancer cells.	Dendritic-Based Neoantigen Vaccines: Ongoing trials in advanced malignant cancers with or without ICIs/chemo (NCT05749627, NCT06675201, NCT04968366, NCT04627246). Sipuleucel-T (Dendreon): Vaccine for mCRPC targeting PAP antigen. Phase I/II trials (NCT06100705; NCT05806814; NCT05751941) actively recruiting. Phase III investigating immune boost response changes in patients with mCRPC (NCT06134232) is active but not recruiting yet (as of the latest update). FDA approved therapy, with proven efficacy in mCRPC over the years [129,130,151]. DOC1021 (Diakonos Oncology): Vaccine for GBM. Phase I/II trial (NCT06805305) recruiting adult GBM patients, evaluating combination with TMZ and tumour resection, with ongoing follow-up on safety and efficacy. DCVax^®^-L (Northwest Biotherapeutics): Vaccine for GBM. Phase I for recurrent ovarian/primary peritoneal cancer (NCT00683241) completed. Expanded Access protocol for GBM patients (NCT02146066) is available for those who failed other trials, with ongoing follow-up on safety and efficacy. Notably, the vaccine shows promise, but clinical outcomes could benefit from further randomised trials to confirm its efficacy [152]. Other ongoing Phase I/II trials targeting multiple cancer types include: IL15-Transpresenting WT1-Targeted Dendritic Cell Vaccine (NCT05964361); HER2-Primed Dendritic Cells (NCT05504707); Dendritic Cell/Tumour Fusion Vaccines (NCT00799110). For information on Dendritic-Based Neoantigen Vaccines, please refer to the Neoantigen Vaccines section.	Strong immune activation by utilising the body’s own dendritic cells to target specific tumour antigens, potentially overcoming immune tolerance and inducing long-term immunity. However, they face limitations related to complex and costly manufacturing processes (patient-specific personalisation adds manufacturing burden), immunosuppressive TME, and variable clinical success, with many trials showing modest outcomes. Additionally, the immune response may not be long-lasting without booster shots [130,151].
Oncolytic Virus Vaccines	Engineered viruses designed to selectively infect and kill cancer cells while stimulating the immune response.	TG4050 (Transgene): Viral-Based Neoantigen Vaccines: Viral vector vaccine in Phase I for HNSCC (NCT04183166); early immunogenicity observed [135,139]. T-VEC (Amgen): First oncolytic viral therapy approved for metastatic melanoma using adenovirus as the viral vector. Ongoing Phase I/II for BC (NCT02779855), melanoma (NCT02965716), sarcoma (NCT04599062; NCT02923778), SCC (NCT04163952) and lymphoma (NCT02978625). Phase III show T-VEC with ICIs resulted in better response rates (CR, ORR, DCR) than T-VEC alone in melanoma (NCT01368276, NCT00769704), with ongoing exploration of combination therapies [153]. RP1 (Replimune): Uses a reovirus. Ongoing phase I/II for solid tumours (NCT04050436). Phase III with Nivo (NCT06264180) for advanced melanoma in patients progressing after prior anti-PD-1/CTLA-4 therapies. Pexa-Vec (SillaJen): Vaccinia virus with additional expression of GM-CSF. Ongoing Phase I/II combined with cemiplimab for RCC (NCT03294083). Phase III completed in HCC (NCT02562755), showing promising results when combined with sorafenib [154].	Targeted tumour destruction, immune activation, and potential for synergistic effects with other therapies. However, limitations include immunity to viral vectors, TME challenges, tumour heterogenicity, limited viral replication, and complex manufacturing, necessitating further research to improve their clinical impact [133].
Viral vector-based vaccines	Viral particles where the genomes have been modified to contain genes coding for cancer antigens.	NOUS-PEV: is a vector-based personalised vaccine, expressing 60 nAgs and consists of priming with a nonhuman Great Ape Adenoviral vector (GAd20), cphase Ib trial of NOUS-PEV in combination with pembrolizumab in treatment-naïve patients with metastatic melanoma (NCT04990479) [74]. PROSTVAC-VF: A Phase II Randomised, Double Blind, Controlled Study to Evaluate the Safety and Efficacy of PROSTVAC^®^-VF/TRICOM™ in Combination with GM-CSF in Patients with Androgen-Independent Adenocarcinoma of the Prostate (NCT00081120)	Induce a robust innate and humoral and cellular immunity, have intrinsic adjuvant properties due to the expression of PAMPs, can be engineered to be replication-competent or replication-deficient allowing for improved safety and reactogenicity [133]

Abbreviations: AA—Amino Acid; AML—Acute Myeloid Leukaemia; BC—Breast Cancer; CIN2/3—Cervical Intraepithelial Neoplasia; Grade 2/3; CR—Complete Response; CRC—Colorectal Cancer; DCIS—Ductal Carcinoma In Situ; DCR—Disease Control Rate; DIPG—Diffuse Intrinsic Pontine Glioma; EBV—Epstein-Barr virus; GBM—Glioblastoma; GM-CSF—Granulocyte-Macrophage Colony-Stimulating Factor; HCC—Hepatocellular Carcinoma; HNSCC—Head and Neck Squamous Cell Carcinoma; ICIs—Immune Checkpoint Inhibitors; mCRPC—Metastatic Castration-Resistant Prostate Cancer; Nivo—Nivolumab; NSCLC—Non-Small Cell Lung Cancer; ORR—Overall Response Rate; PAP—Prostatic Acid Phosphatase; PAMP—pathogen associated molecular patterns; PCa—Prostate Cancer; PDAC—Pancreatic Ductal Adenocarcinoma; Pembro—Pembrolizumab; PFS—Progression-Free Survival; PSA—Prostate-Specific Antigen; RCC—Renal Cell Carcinoma; RT—Radiation Therapy; SCC—Skin Squamous Cell Carcinoma of the skin; TAAs—Tumour-Associated Antigens; TME—Tumour Microenvironment; TMZ—Temozolomide; TNBC—Triple-Negative Breast Cancer; WT1—Wilms Tumour 1. ^1^ All clinical trial identifiers (NCT numbers) listed in this table are registered and can be accessed on ClinicalTrials.gov. Clinical trials were selected based on recent and ongoing trials, and while the list is extensive, it is not exhaustive. Selection was guided by clinical relevance, trial phase, and novelty of therapeutic strategy.

### 3.3. Considerations in the Application of Therapeutic Vaccines in Clinical Settings

Anticancer vaccines are limited clinically by their production, formulation and pharmacokinetic profiles. Formulations can consist of shared antigen vaccines targeting antigens commonly expressed by many cancers (off-the-shelf vaccines) or targeting specific antigens only present on patient tumours (personalised cancer vaccines) [15]. Pharmacokinetic analysis remains a limiting factor; however, for example, personalised vaccines are particularly hindered by a lack of information on their pharmacokinetic profiles due to insufficient dose-finding trials, which limits their clinical assessment. Importantly, formulating a personalised vaccine remains challenging, as it requires a lengthy process of tumour biopsy, genetic analysis, sequencing, and vaccine production and formulation, which may not be effective in an immunocompromised patient with an aggressive tumour [155]. Choosing the optimal antigen for vaccine production is a vital part of vaccine development but is complex due to tumour variations and rapid mutation rates of cancer cells and the heterogeneity of the TME [36]. Vaccine activity in vivo is also impacted by the TME, tumour type, absence of TAAs and TSAs, off-target effects and patient heterogeneity [31,69]. Tumours have an ability to grow in the presence of the immune system, as they have developed immune regulatory mechanisms to enhance their survival, including inflammatory cytokines IL-1 and tumour necrosis factor (TNF) [156]. This immune tolerance and regulation mean the application of immunotherapy in oncology is challenging. Tumours also attract regulatory T cells (Treg), which are suppressive of CD4+ and CD8+ cells, allowing for survival in the TME [157]. DNA vaccines have many advantages, including ease of production and TAAs, but require transcription and translation for uptake by DC cells, may induce off-target immunogenic ADRs and may induce oncogenic activity once integrated into the patient genome [115]. mRNA vaccines are at the forefront of cancer vaccine research, as they have many advantages and have proved efficacy in generating an antitumour response. RNA manufacturing uses a rather simplistic, scalable, and economic cell-free production system [158]. mRNA vaccines induce both innate and adaptive immune responses in the form of activated pattern recognition receptors, e.g., Toll-like receptors (TLRs), and the release of pro-inflammatory cytokines and type 1 IFs, which triggers the adaptive immune response where APCs activate CD4^+^ and CD8^+^ cells, providing an antitumour effect [159]. RNA degradation is a limiting factor, however, affecting the expression and function of genes [123]. mRNA also has issues with instability, in vivo delivery and distribution, and degradation in the TME and relies on specific antigens which may not be present due to tumour heterogeneity [160]. Viral vectors are applied as mRNA gene delivery mechanisms but have issues of limited load capacity, cytotoxicity, immunogenicity, manufacturing difficulties and stability [161]. There may also be antibodies present in the patient to the adenoviral vector, which is most used for vaccine production due to environmental exposure [133]. The use of non-viral vectors, namely lipid nanoparticles (LNPs) or polymers, to deliver the mRNA offers a safe, non-immunogenic, biocompatible, easy-to-produce means of delivering a large gene load to the tumour cells [161]. LNPs are employed to deliver antigens and adjuvants, allowing for directed delivery to APCs, resulting in an elevated immune response [162]. The studies of Oberli et al. (2017) developed an LNP vector to deliver an mRNA vaccine in melanoma models which induced a CD8+ response and tumour shrinkage with uptake into DCs, macrophages, and neutrophils [163]. Studies describe the formulation and efficacy of a lipopolyplex mRNA vaccine which was taken up by DCs, providing an immune response and tumour toxicity in a mice lung cancer model [160]. Non-viral vectors have many advantages and disadvantages as described elsewhere [164]. Studies have described the immune reaction to lipid carrier systems where anaphylactic shock was observed as a potentially fatal outcome [165]. Large-scale production of anti-cancer vaccines using bioprocessing and biological expression systems allows for rapid production, high levels of immunogenicity, biocompatibility and a loss of pathogenicity reversion, which are limitations of traditional vaccine types [93]. The large-scale production of mRNA vaccines and the isolation and purification of the mRNA at downstream processing to achieve clinical purity standards remain challenging, with adherence to Current Good Manufacturing Practice (cGMP) [158]. There is a lack of quality control (QC) standardisation in testing methods applied to mRNA production and formulation. The implementation of a quality by design (QbD) framework in nucleic acid vaccine development with standardised QC procedures in place is required to achieve clinical application [166]. The identification of critical process parameters (CPPs), critical quality attributes (CQAs) and associated acceptance criteria needs to be established in the harmonised design, production, and clinical application of mRNA vaccines [158]. A better understanding of mRNA structure, pro-inflammatory activity in vivo and purity control is needed to allow for widespread application of cancer vaccines clinically. The provision for safe, effective, biocompatible delivery methods for nucleic acid-based therapeutic vaccines is also required to reduce off-target immunogenicity and genotoxic effects [167]. The identification of biomarkers to assess vaccine efficacy would also aid in the clinical application of vaccines, both off-the-shelf and personalised. Long-term studies are scarce, which inhibits efficacy and safety analysis, as tumour cells can mutate quickly, which impacts vaccine efficacy, where booster shots may be required over time [155]. The route of administration is also an important consideration in cancer vaccines, with the intramuscular (IM), subcutaneous, intradermal, intravenous (IV), intratumoural, oral, and mucosal route having direct effects on vaccine efficacy, immune response, and safety. The route of administration is influenced by cancer type, location and vaccine type. For example, peptide vaccines targeting HER2 are administered via the intradermal route due to the presence of a dense network of cutaneous DCs [168]. Studies assessing the optimal route of administration showed that peptide vaccines and poly-ICLC adjuvant administered via the IM route resulted in increased CTL immune responses and antitumour action in comparison to the subcutaneous route in test mice [169]. Establishing the optimal route of administration of cancer vaccine modalities during the pharmacokinetic studies is essential to ensure vaccine efficacy and anti-cancer action in vivo.

Traditional vaccine adjuvants, including mineral salts, aluminum hydroxide, or emulsions, have been applied for decades in preventing infectious disease. Cancer vaccines may also be administered in combination with adjuvants to induce and amplify the host immune response, particularly T-cell immunity. Traditional adjuvants such as alum, however, mainly induce humoral immunity, particularly towards Th2 cells, limiting their efficacy in cancer vaccine immunotherapy, where CD8+ activation is essential for antitumour activity [170]. An ideal cancer vaccine adjuvant should activate T cells, enhance the presentation efficiency of APCs, and contribute to immunity by expressing co-stimulatory molecules induced by APCs. The adjuvants monophosphoryl lipid A (MPL) and cytosine phosphoguanine (CpG), which stimulate toll-like receptors, are currently used in the prophylactic Cervarix™ and Heplisav™ vaccines, respectively [171]. Studies suggest that endogenous adjuvants, including cytokines and TAAs, are released upon tumour cell death, which activates DCs and adaptive immune responses in combination with cancer vaccines. T-vec™ is formulated with a coding sequence of the cytokine GM-CSF, which acts to recruit APCs and CD8+ cells to the tumour [172]. GM-CSF is also widely used as an adjuvant in breast cancer vaccines, where it induces the maturation of myeloid cells, granulocytes and macrophages and promotes the expansion and activation of DCs [168]. Additional adjuvants which are known to activate CD8+ include PolyIC, imiquimod, CpG oligodeoxynucleotides and saponins (e.g., ISCOMATRIX) [172], where CD8 infiltration and penetration are key to antitumour activity. Clinical trials are focusing on the use of a combination of adjuvants such as Montanide ISA-51 and Poly (I: C), a TLR-3 ligand, in terms of efficacy and safety in cancer vaccines and pro-inflammatory cytokines [173]. Recombinant viral vectors used as delivery vehicles also promote immune responses due to TLR and PRR ligands which activate DC and enhance CD8+ cells and limit T cell exhaustion in the TME [168]. Adjuvants used in cancer vaccines are typically classified as immunostimulants, e.g., TAAs or delivery systems. An excellent review of vaccine adjuvants is provided elsewhere [170].

### 3.4. Combination Therapies

Administering cancer vaccines as a combination therapy with radiation, chemotherapy or targeted immunotherapies may provide improved treatment efficacy and patient mortality. Pre-clinical studies show that anti-CTLA-4 ICIs, when used in combination with a cancer vaccine, had improved reduction in tumour size and enhanced antitumour immune responses in mouse models of prostate and melanoma cancers [174]. The mRNA-4157 vaccine, when given in combination with the ICI pembrolizumab, resulted in an increased rate of remission (50%) and prolonged survival in patients with HNSCC in comparison to those receiving pembrolizumab monotherapy [175]. A phase Ib clinical trial assessing the administration of a personalised neoantigen-based vaccine (NEO-PV-01) in combination with PD-1 blockade for the treatment of advanced melanoma, non-small cell lung cancer, or bladder cancer demonstrated safety and an absence of ADRs in 82 patients [176]. Studies have demonstrated the efficacy of mAbs and vaccine combinations in animal and human models where improved survival rates were achieved due to enhanced CD8+ T cell expansion and augmentation and cytokine activity [177].

Vaccines combined with CAR T-cell therapy may aid in overcoming the T-cell exhaustion and reduced cytotoxic activity at the tumour site which hinders CAR T-cell treatment [178]. Vaccines may enhance CAR T cells efficacy by modulating the TME and promoting the expression of TAAs [178]. In radiation therapy the abscopal effect, where systemic tumours are impacted by localised radiation exposure, may allow for enhanced treatment in the presence of cancer vaccines. This abscopal phenomenon is believed to be resultant from systemic immune activation following localised exposure to radiation and has been observed in many solid tumours, e.g., melanoma and renal cell carcinoma [170]. This antitumour activity has been evident in combination approaches using radiation and other immunotherapies with improved patient outcomes [179]. Preclinical studies have demonstrated enhanced antitumour efficacy and abscopal effects in mice treated with radiation in combination with DC vaccines [180]. Initial clinical studies demonstrate the efficacy of administering a peptide vaccine targeting anti-HPV16 in combination with chemotherapy in cases of cervical cancer due to an improved T cell response [175]. Importantly, the application of adjuvants in such combination therapies, including ICIs and mAbs, raises complications in terms of compatibility and the risk of inducing off-target side effects [170]. Such combination approaches may provide improved therapeutic efficacy and aid in overcoming the therapy resistance provided by the TME. Undoubtedly, extensive clinical investigation is warranted to investigate and establish a better comprehensive understanding of the TME, therapeutic resistance and tumour immune escape.

## 4. Advancements Towards Vaccine Efficacy

Improvements in the clinical efficacy of anti-cancer vaccines may be achieved with the implementation of microfluidic technology (MF), machine learning (ML) and artificial intelligence (AI). Current AI databases for cancer vaccine development are epitope databases, including IEDB and SYFPEITHI, and neoantigen peptide databases, dbPepNeo2, and MHC binders, such as MHCBN [181]. AI allows for significant advances in immunotherapy and vaccine development in epitope prediction, for example, where the AI package Disco-Tope 2.0 has been implemented to calculate epitope propensity by analysing varying parameters in vaccine design, with DiscoTope 3.0 advancing on this by integrating a novel learning technique [182]. AI and ML require datasets which must represent the diverse in vivo TME and account for complex biological dynamics to accurately provide computational models which are scalable and precise for vaccine design [183]. This is currently a significant limitation of these methodologies. Quantum machine learning (QML), which is based on the principles of quantum mechanics, may provide benefits in this area, as it can simulate molecular interactions, e.g., binding affinities with exceptional accuracy [183]. The high degree of heterogeneity in individual tumours and the TME are significant obstacles in developing cancer vaccines, as identifying neoantigens which generate an immune response which is tumour toxic is difficult [184]. Advances in genomics, proteomics and gene editing tools in the identification of TAAs and TSAs will allow for better targeting of tumours and decreased off-target ADRs. Gene editing tools such as CRISPR/Cas9 may allow for improved recognition of TAAs and TSAs, increasing specific antitumour activity [185]. CRISPR/Cas9 can detect the function of cancer with high accuracy and efficiency, which may aid in the genomic understanding of cancer and vaccine development when applied for TSA and TAA genes [186]. The application of CRISPR/Cas9 screening technology allows for the identification of genes involved in the immune response in the TME, which can then be targeted with immunotherapy [187]. The application of CRISPR for PD-1 knockdown has shown antitumour efficacy in the TME in FDA-approved CRISPR clinical trials [76]. Potentially fatal immunotoxicity is a risk associated with CRISPR immunotherapy. However, with the FDA acknowledging the risk as top priority in Investigational New Drug (IND) draft [188]. MFs are in vitro assays using microfluidic and tissue engineering advances to investigate disease aetiology by mimicking an in vivo environment, often used in conjunction with ML and omics technology. The research of Huber et al. (2024) used NeoDisc, which is an ML software using AI algorithms to identify TSA HLA-I and II antigens based on genomics and proteomics [189]. The ML NeoaPred was used to construct an MHC peptide–HLA class I complex structure in silico and assess an immunotoxic potential with 82% accuracy [190]. ML has also been applied in optimising polymeric nanoparticles for mRNA delivery in vivo [191]. The development of algorithms predicting the binding of MHC I and II to neoantigens is important in predicting the immunogenicity potential of neoantigens [114]. Unfortunately, ML and AI are greatly hindered by a lack of high-quality data covering a heterogeneous population which is needed to establish accurate algorithms to predict neoantigens, mutations and genomic influences on tumour formation, immune evasion and proliferation [192]. In anti-cancer vaccines, MFs are applied in the development and preparation of LNP delivery systems to improve encapsulation efficiency, in vivo delivery and biocompatibility [167]. The microfluidic Cell Squeeze technology is a novel approach which disrupts the cell membrane to allow for cargo to be delivered inside the cell and has been successful in many immune cells, including DCs, macrophages, T cells and B cells, enabling an MHC I and CD8+ cell activation in animal models [193]. Importantly, cell squeeze technology applied to APCs has shown potent antitumour activity in vivo, which is scalable for clinical use [194]. Studies show that cell squeeze in mice DCs was ca. 1000 times more potent at provoking CD8^+^ T cell responses [195].

## 5. Conclusions

Cancer vaccine immunotherapy modalities offer a novel approach for the prevention and treatment of cancer, to be used in conjunction with traditional oncology methods. Importantly, cancer vaccines can induce the production of new immune cells which are specific for the tumour, giving them an advantage over ICIs therapies, which operate via activation of existing immune cells in the patient. While their potential is clear, cancer vaccines remain challenging for clinical implementation. The TME possesses significant challenges as a complex system consisting of many cell types, inflammatory, immunosuppressive and metabolic variations which impact greatly on immunotherapy modalities. The heterogeneity of tumours and the TME must be fully understood to optimise vaccine formulation and delivery methods to achieve therapeutic efficacy. The identification of biomarkers to assess vaccine efficacy would also aid in the clinical application of vaccines. Novel approaches, including ML and AI, show great potential in identifying important genomic and proteomic alterations in tumour progression but are greatly limited by a lack of relevant data sets.

## Figures and Tables

**Table 1:** Global prevalence, mortality rates and current treatment options (small molecules and biologics) for the 10 most prevalent cancers.

Cancer	Prevalence ^1^ (Millions)	Mortality (Millions) ^1^	Treatment Options ^2^	
			Small Molecules	Biologics
Lung	2.48	1.82	EGFR inhibitors (e.g., erlotinib, gefitinib, osimertinib); ALK inhibitors (e.g., crizotinib, alectinib, brigatinib); ROS1 inhibitors (e.g., crizotinib); RET inhibitors (e.g., selpercatinib, pralsetinib); MET inhibitors (e.g., capmatinib, tepotinib); KRAS G12C inhibitors (e.g., sotorasib); BRAF inhibitors (e.g., dabrafenib); Chemotherapy (e.g., cisplatin, carboplatin); PARP inhibitors (e.g., olaparib, clinical trials only, not approved)	ICIs (e.g., pembrolizumab, nivolumab); CTLA-4 inhibitor (e.g., ipilimumab); Anti-angiogenic mAbs (e.g., bevacizumab, Anti-VEGF).
Breast	2.30	0.66	Hormonal therapy (e.g., tamoxifen, aromatase inhibitors, fulvestrant); CDK4/6 inhibitors (e.g., palbociclib, ribociclib); PI3K inhibitors (e.g., alpelisib); PARP inhibitors (e.g., olaparib for BRCA-mutated breast cancers); AKT inhibitor (e.g., capivasertib for HR+/HER2-negative breast cancer) [16]; Chemotherapy (e.g., taxanes, anthracyclines); Oral SERDs (e.g., elacestrant, emerging, not approved, under investigation) [17].	Anti-HER2 mAbs (e.g., trastuzumab); HER2 ADCs (e.g., T-DM1, T-DXd); ICIs (e.g., atezolizumab, pembrolizumab).
Colorectal	1.93	0.90	BRAF inhibitors (e.g., encorafenib); MKI (e.g., regorafenib); HER2 inhibitors (e.g., tucatinib, approved only in combination with the biologic trastuzumab for HER2-positive mCRC); Chemotherapy (e.g., 5-FU, oxaliplatin, irinotecan); Clinical trials ongoing for other HER2 inhibitors (e.g., lapatinib, under investigation, not approved) [18].	Anti-angiogenic mAbs (e.g., bevacizumab, Anti-VEGF); ICIs (e.g., pembrolizumab, nivolumab); HER2-targeted antibodies (e.g., trastuzumab + tucatinib, first FDA-approved combo for HER2+ mCRC); Other HER2 biologics are in trials [18]
Prostate	1.47	0.40	Androgen receptor inhibitors (e.g., enzalutamide); Androgen biosynthesis inhibitor (e.g., abiraterone acetate); CYP17 inhibitors; PARP inhibitors (e.g., olaparib, rucaparib for BRCA/HRR-mutated mCRPC); Chemotherapy (e.g., docetaxel, cabazitaxel)	Therapeutic vaccine (i.e., Sipuleucel-T); Immunotherapy (e.g., pembrolizumab, approved only in certain cases, i.e., mCRPC).
Stomach	0.97	0.66	Chemotherapy (e.g., 5-FU, capecitabine, cisplatin); MKI (e.g., regorafenib, approved for advanced/refractory GC); HER2 inhibitors (e.g., lapatinib, clinical trials only, not approved) [19].	HER2-targeted mAb (e.g., trastuzumab for HER2+ cases); Anti-VEGFR2 mAb (e.g., ramucirumab, approved for advanced disease); ICIs (e.g., nivolumab, pembrolizumab)
Liver	0.87	0.76	MKI/TKI (e.g., sorafenib, regorafenib, lenvatinib); MET inhibitor (e.g., tepotinib, clinical trials only, not approved) [20,21].	ICIs (PD-1 inhibitors, e.g., atezolizumab + bevacizumab, durvalumab, pembrolizumab, and CTLA-4 inhibitor, e.g., ipilimumab with nivolumab); Anti-angiogenic mAb (e.g., bevacizumab, Anti-VEGF).
Thyroid	0.82	0.048	MKI (e.g., sorafenib, lenvatinib); RET inhibitors (e.g., selpercatinib, pralsetinib); BRAF inhibitors (e.g., dabrafenib, approved for BRAF V600E-mutant ATC, usually with the MEK inhibitor, trametinib); ALK inhibitors (e.g., larotrectinib, entrectinib for NTRK fusion-positive thyroid cancers, very rare, tissue-agnostic approval) [22].	No biologics are approved as standard first-line treatments for thyroid cancer; ICIs (e.g., pembrolizumab) are approved only for rare cases with specific biomarkers (e.g., MSI-H, TMB-high, or PD-L1 positive).
Cervical	0.66	0.35	Chemotherapy (e.g., cisplatin, paclitaxel); MKIs (e.g., pazopanib, clinical trials only, not standard) [23]	Anti-angiogenic mAb (e.g., bevacizumab); ICIs (e.g., pembrolizumab for PD-L1 positive recurrent/metastatic cervical cancer); ADCs (e.g., tisotumab vedotin); Other biologics under clinical investigation include ICIs (e.g., nivolumab, ipilimumab), therapeutic vaccines (e.g., ADXS11-001, ISA101), EGFR antibodies, and CAR T therapies [24].
Bladder	0.61	0.22	FGFR inhibitors (e.g., erdafitinib); Traditional chemotherapies (e.g., cisplatin, gemcitabine, carboplatin, methotrexate); MTIs (e.g., vinflunine, EMA-approved for mTCCU); Other small molecules in trials include TYRA-300 [25], an FGFR3 inhibitor, in phase 1 for low-grade NMIBC and advanced urothelial carcinoma.	ICIs (e.g., pembrolizumab, atezolizumab, nivolumab); ADCs (e.g., enfortumab vedotin); IL-15 receptor agonist (e.g., Anktiva); Other biologics under clinical investigation for bladder cancer include anti-VEGF monoclonal antibody (bevacizumab) [10]; ADCs (with PD-inhibitors) [26,27,28].
Non-Hodgkin lymphoma	0.55	0.25	BTK inhibitors (e.g., ibrutinib, acalbrutinib; BCL-2 inhibitors (e.g., venetoclax); PI3K inhibitors (e.g., idelalisib, duvelisib, copanlisib); Chemotherapy (e.g., cyclophosphamide, doxorubicin, vincristine).	Anti-CD20 mAbs (e.g., rituximab, obinutuzumab); CAR T-cell therapy (e.g., axicabtagene ciloleucel); Immunomodulatory agents (e.g., lenalidomide); ICIs (e.g., pembrolizumab, nivolumab); ADCs (e.g., brentuximab vedotin); Radiolabeled antibodies (e.g., ibritumomab tiuxetan); T-cell engagers (e.g., blinatumomab for relapsed/refractory B-cell NHL); Rituximab biosimilars (e.g., truxima, ruxience, riabni); Other biologics in trials include IL-2 cytokine therapy.

Abbreviations: ADC—Antibody Drug Conjugate; ALK—Anaplastic Lymphoma Kinase; ATC—Anaplastic Thyroid Cancer; BRCA—Breast Cancer gene; BTK—Bruton’s tyrosine kinase; EGFR—Epidermal Growth Factor Receptor; FGFR3—Fibroblast Growth Factor Receptor 3; HRR—Homologous Recombination Repair; ICIs—Immune Checkpoint Inhibitors; IL-2—Interleukin-2; KRAS—Kirsten Rat Sarcoma Virus; mAb—monoclonal antibody; mCRPC—Metastatic Castration-Resistant Prostate Cancer; MET—Mesenchymal Epithelial Transition; MKI—Multi-Kinase Inhibitor; MSI-H—Microsatellite Instability-High and Deficient Mismatch Repair; mTCCU—Metastatic Transitional Cell Carcinoma of the Urothelial Tract; MTIs—Microtubule Inhibitors; NHL—Non-Hodgkin Lymphoma; PARP—Poly(ADP-ribose) Polymerase; RET—Rearranged during Transfection; SERDs—Selective Estrogen Receptor Degraders; T-DM1—Trastuzumab Emtansine; T-DXd—Trastuzumab Deruxtecan; TKI—Tyrosine Kinase Inhibitors; TMB—Tumour Mutational Burden; VEGF—Vascular Endothelial Growth Factor; 5-Fu—5-Fluorouracil. ^1^ Global prevalence and mortality rates were sourced were from the WHO’s International Agency for Research on Cancer (IARC), specifically from the ‘Global Cancer Observatory’, using the most recent data available (2022). Figures are presented in millions per year. ^2^ Treatment information was sourced from reputable organisations, including the National Cancer Institute (NCI), World Health Organisation (WHO), and other authoritative bodies (e.g., EMA, FDA, ESMO, IARC). Treatment options shown are representative, not exhaustive, and selected based on current standards of care and approved agents, with emerging treatment options also included.

**Table 2:** How cancer vaccines may overcome the limitations of other immunotherapy types.

Immunotherapy Type	Limitations	How Vaccines May Overcome Limitations
Immune Checkpoint Inhibitor (ICI)	Limited response rates and development of acquired resistance; immune-related adverse events (irAEs); biomarker identification challenges; high cost and inequitable global access [78].	Cancer vaccines enhance tumour immunogenicity by expanding and diversifying tumour-specific T-cell responses, including memory and effector functions [79,80,81]. They help overcome resistance to ICIs by promoting T-cell infiltration and modulating immunosuppressive features within the TME [78,79,82]. Vaccines typically induce more targeted immune activation, which may contribute to a reduced incidence of immune-related adverse events compared to systemic checkpoint blockade therapies [80,82]. Furthermore, many vaccine platforms are cost-effective and scalable, offering potential for broader global accessibility [78,82].
Antibody-Drug Conjugate (ADC)	Neuropathy and ocular toxicity; heterogeneous antigen expression limiting efficacy; resistance, including multidrug efflux mechanisms; delivery and premature payload release causing off-target toxicities [83].	Cancer vaccines elicit immune-mediated tumour killing without cytotoxic payloads, target multiple tumour antigens to reduce resistance from antigen heterogeneity, and promote durable memory T-cell responses with minimal off-target toxicity [78,79,84].
Bispecific Antibody	Tumour antigen heterogeneity; intractable TME; limited T-cell activation; systemic toxicities such as cytokine release syndrome (CRS); off-target effects damaging healthy tissues; need for frequent dosing; manufacturing complexity and immunogenicity risk [84].	Cancer vaccines induce broad, polyclonal T-cell responses targeting diverse tumour antigens, addressing heterogeneity and immune escape [78,79,80] while minimising systemic toxicities; they also activate and sustain T cells within the TME for durable immunity [80,82]. Recent preclinical studies show that tumour-specific and non-specific vaccination prior to CD3 bispecific antibody therapy enhances T-cell infiltration, polarises the TME toward a pro-inflammatory state, improves therapeutic efficacy, and establishes durable immune memory in both ‘cold’ and ‘hot’ tumours [85,86].
IL-15 Superagonist Cytokine Therapy	Toxicity, including CRS and organ inflammation; short in vivo half-life requiring complex dosing; immunosuppressive TME limiting efficacy; lack of tumour-specific targeting causing systemic immune activation; and potential immune cell exhaustion reducing long-term responses [87].	Cancer vaccines induce tumour-specific T-cell responses that localise immunity to the tumour, reducing systemic toxicity seen with IL-15 superagonists. They promote durable memory T cells resistant to exhaustion and improve antigen presentation to counteract tumour immunosuppression [88]. While vaccines address key IL-15 limitations independently, combining both may further enhance antitumour efficacy [89].
Tumour-Infiltrating Lymphocyte (TIL) Therapy	Manufacturing complexity and variability; limited TIL expansion from some tumours; high cost and labour-intensive process; patient conditioning toxicity; potential for T-cell exhaustion and limited persistence [90].	Cancer vaccines can prime and expand tumour-specific T cells in vivo without the need for ex vivo cell manipulation, enabling more consistent and scalable immune responses. They also promote durable memory T-cell formation and can enhance antigen spreading to target tumour heterogeneity, potentially reducing T-cell exhaustion and improving persistence [88]. By converting ‘cold’ tumours into ‘hot’ ones, they increase T-cell infiltration and function, offering a complementary strategy to TIL therapy [91].

## Data Availability

Data sharing not applicable.
